# Crystal structure of bis­(η^5^-cyclo­penta­dien­yl)(2,3-di­ethyl­butane-1,4-di­yl)hafnium(IV)

**DOI:** 10.1107/S2056989014026929

**Published:** 2015-01-01

**Authors:** Vladimir V. Burlakov, Wolfgang Baumann, Perdita Arndt, Anke Spannenberg, Uwe Rosenthal

**Affiliations:** a A. N. Nesmeyanov Institute of Organoelement Compounds, Russian Academy of Sciences, Vavilov St 28, 119991 Moscow, Russia; b Leibniz-Institut für Katalyse e. V. an der Universität Rostock, Albert-Einstein-Strasse 29a, 18059 Rostock, Germany

**Keywords:** crystal structure, hafnocene, five-membered metallacycle

## Abstract

The title compound, [Hf(C_5_H_5_)_2_(C_8_H_16_)], proves a structural motif of hafna­cyclo­pentane besides the coordination of two cyclo­penta­dienyl ligands in an η^5^-fashion. The hafna­cyclo­pentane ring has a twist conformation and is substituted by two ethyl groups in the β,β′-positions, which are *trans* orientated to each other. One cyclo­penta­dienyl ring and one ethyl group are each disordered over two positions with site-occupancy ratios of 0.679 (15):0.321 (15) and 0.702 (18):0.298 (18), respectively.

## Related literature   

For crystal structures of unsubstituted metalla­cyclo­pentane complexes of group 4 metallocenes, see: Beweries, Fischer *et al.* (2009 ▸); Mansel *et al.* (1997 ▸); Takahashi *et al.* (1996 ▸); Klahn *et al.* (2009 ▸); McGovern *et al.* (2012 ▸); Lee *et al.* (1999 ▸). For crystal structures of 2,4-phenyl­substituted metalla­cyclo­pentane complexes of group 4 metallocenes, see: Beweries, Burlakov *et al.* (2009 ▸); Mansel *et al.* (1997 ▸).
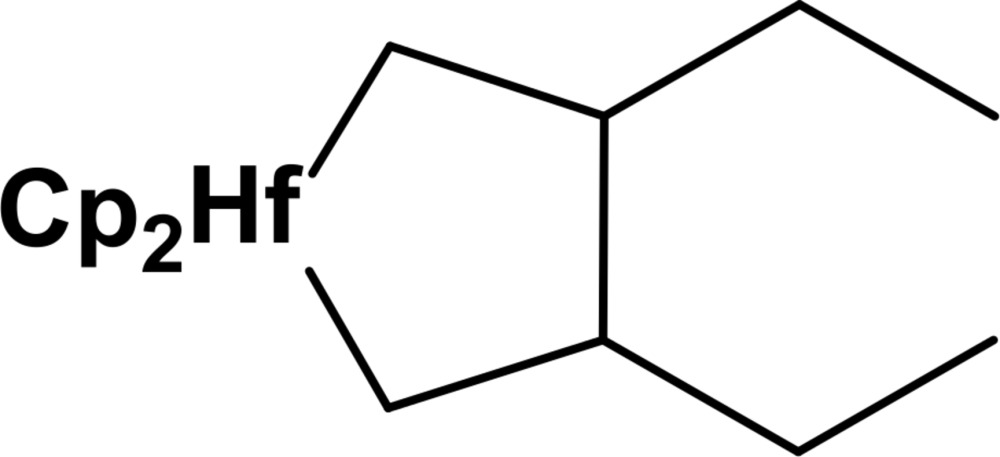


## Experimental   

### Crystal data  

[Hf(C_5_H_5_)_2_(C_8_H_16_)]
*M* *_r_* = 420.88
Monoclinic, 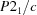

*a* = 12.7055 (6) Å
*b* = 15.5909 (5) Å
*c* = 8.1035 (3) Å
β = 93.982 (3)°
*V* = 1601.35 (11) Å^3^
*Z* = 4
Mo *K*α radiation
μ = 6.50 mm^−1^
*T* = 200 K
0.50 × 0.48 × 0.15 mm

### Data collection  

Stoe IPDS II diffractometer
Absorption correction: numerical (*X-SHAPE* and *X-RED32*; Stoe & Cie, 2005 ▸) *T*_min_ = 0.157, *T*_max_ = 0.361
25548 measured reflections
3679 independent reflections
3120 reflections with *I* > 2σ(*I*)
*R*_int_ = 0.033

### Refinement  

*R*[*F* ^2^ > 2σ(*F* ^2^)] = 0.032
*wR*(*F* ^2^) = 0.069
*S* = 1.15
3679 reflections
193 parameters
64 restraints
H-atom parameters constrained
Δρ_max_ = 1.51 e Å^−3^
Δρ_min_ = −1.55 e Å^−3^

### 

Data collection: *X-AREA* (Stoe & Cie, 2005 ▸); cell refinement: *X-AREA*; data reduction: *X-AREA*; program(s) used to solve structure: *SHELXS97* (Sheldrick, 2008 ▸); program(s) used to refine structure: *SHELXL2014* (Sheldrick, 2008 ▸); molecular graphics: *XP* in *SHELXTL* (Sheldrick, 2008 ▸); software used to prepare material for publication: *SHELXL2014*.

## Supplementary Material

Crystal structure: contains datablock(s) I, New_Global_Publ_Block. DOI: 10.1107/S2056989014026929/is5384sup1.cif

Structure factors: contains datablock(s) I. DOI: 10.1107/S2056989014026929/is5384Isup2.hkl

**Fig. 1.**
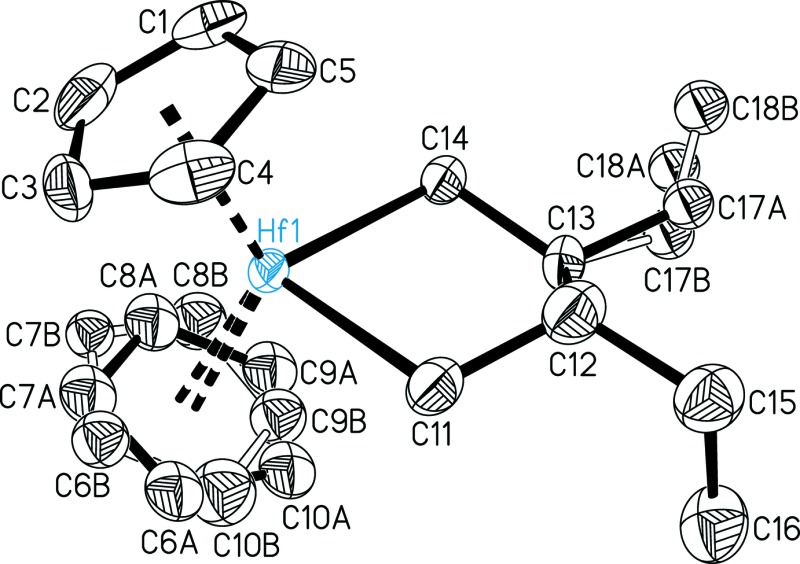
Molecular structure of the title compound with atom labelling and displacement ellipsoids drawn at 30% probability level. H atoms have been omitted for clarity.

. DOI: 10.1107/S2056989014026929/is5384fig1.tif
Mol­ecular structure of the title compound with atom labelling and displacement ellipsoids drawn at 30% probability level. H atoms have been omitted for clarity.

CCDC reference: 1038060

Additional supporting information:  crystallographic information; 3D view; checkCIF report

## supplementary crystallographic information

### S1. Synthesis and crystallization

A suspension of Cp_2_HfCl_2_ (2.243 g, 5.91 mmol) in 20 ml of toluene was treated with 7.5 ml (12.0 mmol) of a 1.6 M solution of *n*-BuLi in *n*-hexane. The mixture was stirred for 30 minutes at room temperature. After filtration bis­(tri­methyl­silyl)acetyl­ene (1.5 ml, 6.67 mmol) and pyridine (0.60 ml, 7.45 mmol) were added to the resulting yellow solution. The reaction mixture was stirred 3.5 hours at 100 °C. All volatiles were evaporated from the dark purple solution and the residue was extracted with 40–50 ml of *n*-hexane at 55 °C. The solution was filtered, concentrated in vacuum to 10–15 ml and stored at –78°C. After one day dark purple crystals had formed which were isolated by decanting of the mother liquor, washed with cold *n*-hexane and dried in vacuum to give a mixture of the alkyne complex Cp_2_Hf(η^2^-Me_3_SiC_2_SiMe_3_)(py) and the title compound in a ratio of 2:1 (checked by NMR). After recrystallization from *n*-hexane brown crystals of the title complex were isolated: 0,120 g, yield: 5 %. Anal. Calcd. for C_18_H_26_Hf (420.89 g·mol^-1^): C 51.37, H 6.23%. Found: C 50.99, H 6.03%.

Single Crystals were obtained from a saturated solution (*n*-hexane) at ambient temperature.

### S2. Refinement

H atoms were placed in idealized positions with *d*(C—H) = 0.95–1.00 Å (CH), 0.99 Å (CH_2_) and 0.98 Å (CH_3_), and refined using a riding model with *U*_iso_(H) fixed at 1.2 *U*_eq_(C) for CH and CH_2_ and 1.5 *U*_eq_(C) for CH_3_. *SADI* and *SAME* instructions were used to improve the geometry of the cyclo­penta­dienyl rings. Additionally, the anisotropic displacement parameters of C6A–C10A were restrained to be equal (*SIMU*). *SADI* was used for the disordered ethyl group. For the ethyl group C15, C16 the use of *DFIX* was necessary due to unresolved disorder. Atoms of the disordered ethyl group and the minor occupied atoms of the disordered cyclo­penta­dienyl ring are refined isotropically. The highest peak in the final difference Fourier map is located 1.36 Å from C13 and the deepest hole 0.83 Å from C3.

### Crystal data

**Table:**

[Hf(C_5_H_5_)_2_(C_8_H_16_)]	*F*(000) = 824
*M**_r_* = 420.88	*D*_x_ = 1.746 Mg m^−^^3^
Monoclinic, *P*2_1_/*c*	Mo *K*α radiation, λ = 0.71073 Å
*a* = 12.7055 (6) Å	Cell parameters from 7164 reflections
*b* = 15.5909 (5) Å	θ = 2.0–29.6°
*c* = 8.1035 (3) Å	µ = 6.50 mm^−^^1^
β = 93.982 (3)°	*T* = 200 K
*V* = 1601.35 (11) Å^3^	Prism, colourless
*Z* = 4	0.50 × 0.48 × 0.15 mm

### Data collection

**Table:**

Stoe IPDS II diffractometer	3679 independent reflections
Radiation source: fine-focus sealed tube	3120 reflections with *I* > 2σ(*I*)
Graphite monochromator	*R*_int_ = 0.033
ω scans	θ_max_ = 27.5°, θ_min_ = 2.1°
Absorption correction: numerical (*X-SHAPE* and *X-RED32*; Stoe & Cie, 2005)	*h* = −16→16
*T*_min_ = 0.157, *T*_max_ = 0.361	*k* = −20→20
25548 measured reflections	*l* = −10→10

### Refinement

**Table:**

Refinement on *F*^2^	64 restraints
Least-squares matrix: full	Hydrogen site location: inferred from neighbouring sites
*R*[*F*^2^ > 2σ(*F*^2^)] = 0.032	H-atom parameters constrained
*wR*(*F*^2^) = 0.069	*w* = 1/[σ^2^(*F*_o_^2^) + (0.0254*P*)^2^ + 4.0826*P*] where *P* = (*F*_o_^2^ + 2*F*_c_^2^)/3
*S* = 1.15	(Δ/σ)_max_ = 0.001
3679 reflections	Δρ_max_ = 1.51 e Å^−^^3^
193 parameters	Δρ_min_ = −1.55 e Å^−^^3^

### Special details

**Table:**

Experimental. ^1^H NMR (400 MHz, C_6_D_6_, 297 K): 0.30 (dd, 2H, Hf-CH_2_); 1.02 (t, 6H, CH_3_); 1.14 (m, 2H, CH_2_); 1.18 (dd, 2H, Hf-CH_2_); 1.60 (m, 2H, CH); 1.78 (m, 2H, CH_2_); 5.79 (s, 10H, Cp).^13^C NMR (100 MHz, C_6_D_6_, 297 K): 11.0 (CH_3_); 31.7 (CH_2_), 45.8 (CH), 50.9 (Hf-CH_2_), 110.8 (Cp).
Geometry. All e.s.d.'s (except the e.s.d. in the dihedral angle between two l.s. planes) are estimated using the full covariance matrix. The cell e.s.d.'s are taken into account individually in the estimation of e.s.d.'s in distances, angles and torsion angles; correlations between e.s.d.'s in cell parameters are only used when they are defined by crystal symmetry. An approximate (isotropic) treatment of cell e.s.d.'s is used for estimating e.s.d.'s involving l.s. planes.

### Fractional atomic coordinates and isotropic or equivalent isotropic displacement parameters (Å^2^)

**Table:**

	*x*	*y*	*z*	*U*_iso_*/*U*_eq_	Occ. (<1)
C1	0.8579 (5)	0.5055 (4)	−0.1657 (8)	0.0641 (18)	
H1	0.9276	0.5267	−0.1441	0.077*	
C2	0.8303 (6)	0.4204 (4)	−0.1904 (8)	0.073 (2)	
H2	0.8773	0.3729	−0.1868	0.088*	
C3	0.7219 (6)	0.4168 (4)	−0.2213 (8)	0.072 (2)	
H3	0.6814	0.3665	−0.2439	0.087*	
C4	0.6830 (5)	0.4995 (4)	−0.2133 (8)	0.0659 (19)	
H4	0.6110	0.5157	−0.2296	0.079*	
C5	0.7669 (4)	0.5545 (4)	−0.1778 (8)	0.0593 (17)	
H5	0.7628	0.6149	−0.1641	0.071*	
C6A	0.6598 (9)	0.3385 (6)	0.1957 (12)	0.063 (2)	0.679 (15)
H6A	0.5865	0.3318	0.1671	0.075*	0.679 (15)
C7A	0.7430 (9)	0.3033 (6)	0.1132 (12)	0.063 (2)	0.679 (15)
H7A	0.7356	0.2671	0.0189	0.075*	0.679 (15)
C8A	0.8388 (9)	0.3302 (6)	0.1926 (13)	0.064 (2)	0.679 (15)
H8A	0.9072	0.3168	0.1596	0.077*	0.679 (15)
C9A	0.8161 (8)	0.3803 (6)	0.3292 (12)	0.063 (2)	0.679 (15)
H9A	0.8660	0.4058	0.4072	0.076*	0.679 (15)
C10A	0.7060 (8)	0.3858 (6)	0.3289 (11)	0.062 (2)	0.679 (15)
H10A	0.6685	0.4167	0.4070	0.074*	0.679 (15)
C6B	0.6940 (15)	0.3112 (15)	0.115 (3)	0.058 (6)*	0.321 (15)
H6B	0.6423	0.2903	0.0350	0.070*	0.321 (15)
C7B	0.8033 (15)	0.3073 (12)	0.111 (2)	0.045 (5)*	0.321 (15)
H7B	0.8393	0.2798	0.0267	0.054*	0.321 (15)
C8B	0.8529 (15)	0.3498 (17)	0.248 (3)	0.069 (8)*	0.321 (15)
H8B	0.9266	0.3577	0.2704	0.083*	0.321 (15)
C9B	0.7724 (19)	0.3784 (16)	0.345 (3)	0.064 (7)*	0.321 (15)
H9B	0.7815	0.4092	0.4464	0.077*	0.321 (15)
C10B	0.6771 (17)	0.353 (2)	0.265 (3)	0.085 (9)*	0.321 (15)
H10B	0.6098	0.3627	0.3063	0.102*	0.321 (15)
C11	0.6206 (5)	0.5450 (4)	0.1446 (9)	0.0581 (15)	
H11A	0.5699	0.5547	0.0481	0.070*	
H11B	0.5828	0.5167	0.2326	0.070*	
C12	0.6663 (5)	0.6305 (4)	0.2073 (9)	0.0670 (18)	
H12	0.6837	0.6629	0.1062	0.080*	
C13	0.7739 (4)	0.6145 (3)	0.3070 (7)	0.0437 (12)	
H13A	0.7572	0.5775	0.4026	0.052*	0.702 (18)
H13B	0.7510	0.5680	0.3816	0.052*	0.298 (18)
C14	0.8524 (5)	0.5617 (4)	0.2094 (8)	0.0505 (14)	
H14A	0.9068	0.5346	0.2859	0.061*	
H14B	0.8878	0.5989	0.1312	0.061*	
C15	0.5878 (6)	0.6866 (4)	0.2924 (8)	0.074 (2)	
H15A	0.6193	0.7438	0.3149	0.089*	
H15B	0.5235	0.6943	0.2177	0.089*	
C16	0.5580 (7)	0.6471 (5)	0.4531 (8)	0.083 (2)	
H16A	0.5294	0.5895	0.4317	0.125*	
H16B	0.5047	0.6829	0.5013	0.125*	
H16C	0.6207	0.6434	0.5303	0.125*	
C17A	0.8212 (6)	0.6962 (5)	0.3831 (12)	0.048 (2)*	0.702 (18)
H17A	0.7703	0.7219	0.4560	0.057*	0.702 (18)
H17B	0.8324	0.7378	0.2937	0.057*	0.702 (18)
C18A	0.9256 (7)	0.6813 (6)	0.4832 (12)	0.056 (3)*	0.702 (18)
H18A	0.9522	0.7360	0.5290	0.083*	0.702 (18)
H18B	0.9148	0.6412	0.5737	0.083*	0.702 (18)
H18C	0.9770	0.6571	0.4113	0.083*	0.702 (18)
C17B	0.8322 (14)	0.6707 (13)	0.4368 (19)	0.051 (6)*	0.298 (18)
H17C	0.8474	0.6334	0.5345	0.061*	0.298 (18)
H17D	0.7802	0.7136	0.4696	0.061*	0.298 (18)
C18B	0.9336 (15)	0.7204 (14)	0.415 (3)	0.060 (7)*	0.298 (18)
H18D	0.9545	0.7514	0.5170	0.090*	0.298 (18)
H18E	0.9897	0.6804	0.3893	0.090*	0.298 (18)
H18F	0.9217	0.7614	0.3237	0.090*	0.298 (18)
Hf1	0.75401 (2)	0.46108 (2)	0.07167 (2)	0.03553 (7)	

### Atomic displacement parameters (Å^2^)

**Table:**

	*U*^11^	*U*^22^	*U*^33^	*U*^12^	*U*^13^	*U*^23^
C1	0.057 (4)	0.096 (5)	0.042 (3)	−0.001 (4)	0.019 (3)	0.012 (3)
C2	0.102 (6)	0.078 (5)	0.044 (4)	0.023 (5)	0.027 (4)	−0.001 (3)
C3	0.124 (7)	0.049 (4)	0.045 (4)	−0.030 (4)	0.013 (4)	−0.013 (3)
C4	0.054 (4)	0.097 (5)	0.046 (4)	0.007 (4)	−0.001 (3)	0.022 (4)
C5	0.075 (4)	0.056 (4)	0.048 (3)	−0.005 (3)	0.015 (3)	0.019 (3)
C6A	0.090 (5)	0.047 (3)	0.052 (3)	−0.007 (3)	0.008 (3)	0.013 (2)
C7A	0.090 (5)	0.045 (3)	0.053 (3)	−0.004 (3)	0.007 (3)	0.011 (2)
C8A	0.091 (5)	0.047 (3)	0.054 (3)	−0.001 (3)	0.006 (3)	0.012 (2)
C9A	0.090 (5)	0.048 (3)	0.051 (3)	−0.002 (3)	0.006 (3)	0.014 (2)
C10A	0.089 (5)	0.048 (3)	0.050 (3)	−0.005 (3)	0.009 (3)	0.014 (2)
C11	0.050 (3)	0.065 (4)	0.060 (4)	0.000 (3)	0.014 (3)	−0.014 (3)
C12	0.070 (4)	0.067 (4)	0.066 (4)	0.013 (4)	0.016 (3)	0.005 (3)
C13	0.055 (3)	0.037 (2)	0.039 (3)	0.010 (2)	0.001 (2)	−0.003 (2)
C14	0.046 (3)	0.050 (3)	0.057 (4)	−0.004 (2)	0.015 (3)	−0.014 (3)
C15	0.072 (5)	0.076 (5)	0.075 (5)	0.010 (4)	0.011 (4)	0.007 (4)
C16	0.109 (7)	0.083 (5)	0.058 (5)	0.005 (5)	0.005 (4)	−0.005 (4)
Hf1	0.04715 (12)	0.03087 (10)	0.02962 (10)	−0.00094 (11)	0.01014 (7)	−0.00135 (9)

### Geometric parameters (Å, º)

**Table:**

C1—C2	1.383 (4)	C8B—H8B	0.9500
C1—C5	1.384 (4)	C9B—C10B	1.390 (17)
C1—Hf1	2.505 (6)	C9B—Hf1	2.56 (3)
C1—H1	0.9500	C9B—H9B	0.9500
C2—C3	1.383 (4)	C10B—Hf1	2.54 (3)
C2—Hf1	2.477 (6)	C10B—H10B	0.9500
C2—H2	0.9500	C11—C12	1.527 (9)
C3—C4	1.383 (4)	C11—Hf1	2.253 (6)
C3—Hf1	2.478 (6)	C11—H11A	0.9900
C3—H3	0.9500	C11—H11B	0.9900
C4—C5	1.383 (4)	C12—C15	1.526 (2)
C4—Hf1	2.493 (6)	C12—C13	1.560 (9)
C4—H4	0.9500	C12—H12	1.0000
C5—Hf1	2.506 (6)	C13—C17A	1.521 (8)
C5—H5	0.9500	C13—C17B	1.522 (8)
C6A—C10A	1.401 (5)	C13—C14	1.552 (7)
C6A—C7A	1.402 (5)	C13—H13A	1.0000
C6A—Hf1	2.502 (9)	C13—H13B	1.0000
C6A—H6A	0.9500	C14—Hf1	2.253 (6)
C7A—C8A	1.401 (5)	C14—H14A	0.9900
C7A—Hf1	2.488 (10)	C14—H14B	0.9900
C7A—H7A	0.9500	C15—C16	1.511 (2)
C8A—C9A	1.401 (5)	C15—H15A	0.9900
C8A—Hf1	2.478 (10)	C15—H15B	0.9900
C8A—H8A	0.9500	C16—H16A	0.9800
C9A—C10A	1.401 (5)	C16—H16B	0.9800
C9A—Hf1	2.517 (10)	C16—H16C	0.9800
C9A—H9A	0.9500	C17A—C18A	1.524 (10)
C10A—Hf1	2.504 (9)	C17A—H17A	0.9900
C10A—H10A	0.9500	C17A—H17B	0.9900
C6B—C7B	1.392 (16)	C18A—H18A	0.9800
C6B—C10B	1.410 (17)	C18A—H18B	0.9800
C6B—Hf1	2.49 (2)	C18A—H18C	0.9800
C6B—H6B	0.9500	C17B—C18B	1.524 (10)
C7B—C8B	1.404 (17)	C17B—H17C	0.9900
C7B—Hf1	2.492 (18)	C17B—H17D	0.9900
C7B—H7B	0.9500	C18B—H18D	0.9800
C8B—C9B	1.407 (17)	C18B—H18E	0.9800
C8B—Hf1	2.53 (3)	C18B—H18F	0.9800
			
C2—C1—C5	108.5 (5)	C17A—C13—H13A	105.5
C2—C1—Hf1	72.8 (4)	C14—C13—H13A	105.5
C5—C1—Hf1	74.0 (3)	C12—C13—H13A	105.5
C2—C1—H1	125.7	C17B—C13—H13B	98.5
C5—C1—H1	125.7	C14—C13—H13B	98.5
Hf1—C1—H1	119.2	C12—C13—H13B	98.5
C1—C2—C3	107.7 (6)	C13—C14—Hf1	105.5 (4)
C1—C2—Hf1	75.0 (4)	C13—C14—H14A	110.6
C3—C2—Hf1	73.8 (4)	Hf1—C14—H14A	110.6
C1—C2—H2	126.1	C13—C14—H14B	110.6
C3—C2—H2	126.1	Hf1—C14—H14B	110.6
Hf1—C2—H2	117.1	H14A—C14—H14B	108.8
C4—C3—C2	108.0 (5)	C16—C15—C12	111.5 (6)
C4—C3—Hf1	74.4 (4)	C16—C15—H15A	109.3
C2—C3—Hf1	73.8 (4)	C12—C15—H15A	109.3
C4—C3—H3	126.0	C16—C15—H15B	109.3
C2—C3—H3	126.0	C12—C15—H15B	109.3
Hf1—C3—H3	117.8	H15A—C15—H15B	108.0
C3—C4—C5	108.4 (5)	C15—C16—H16A	109.5
C3—C4—Hf1	73.3 (4)	C15—C16—H16B	109.5
C5—C4—Hf1	74.5 (3)	H16A—C16—H16B	109.5
C3—C4—H4	125.8	C15—C16—H16C	109.5
C5—C4—H4	125.8	H16A—C16—H16C	109.5
Hf1—C4—H4	118.4	H16B—C16—H16C	109.5
C4—C5—C1	107.4 (5)	C13—C17A—C18A	113.1 (7)
C4—C5—Hf1	73.4 (3)	C13—C17A—H17A	109.0
C1—C5—Hf1	73.9 (3)	C18A—C17A—H17A	109.0
C4—C5—H5	126.3	C13—C17A—H17B	109.0
C1—C5—H5	126.3	C18A—C17A—H17B	109.0
Hf1—C5—H5	118.4	H17A—C17A—H17B	107.8
C10A—C6A—C7A	106.4 (8)	C17A—C18A—H18A	109.5
C10A—C6A—Hf1	73.8 (5)	C17A—C18A—H18B	109.5
C7A—C6A—Hf1	73.1 (6)	H18A—C18A—H18B	109.5
C10A—C6A—H6A	126.8	C17A—C18A—H18C	109.5
C7A—C6A—H6A	126.8	H18A—C18A—H18C	109.5
Hf1—C6A—H6A	118.4	H18B—C18A—H18C	109.5
C8A—C7A—C6A	108.9 (8)	C13—C17B—C18B	125.9 (14)
C8A—C7A—Hf1	73.2 (6)	C13—C17B—H17C	105.8
C6A—C7A—Hf1	74.2 (5)	C18B—C17B—H17C	105.8
C8A—C7A—H7A	125.5	C13—C17B—H17D	105.8
C6A—C7A—H7A	125.5	C18B—C17B—H17D	105.8
Hf1—C7A—H7A	118.8	H17C—C17B—H17D	106.2
C9A—C8A—C7A	108.0 (8)	C17B—C18B—H18D	109.5
C9A—C8A—Hf1	75.2 (5)	C17B—C18B—H18E	109.5
C7A—C8A—Hf1	74.0 (6)	H18D—C18B—H18E	109.5
C9A—C8A—H8A	126.0	C17B—C18B—H18F	109.5
C7A—C8A—H8A	126.0	H18D—C18B—H18F	109.5
Hf1—C8A—H8A	116.8	H18E—C18B—H18F	109.5
C10A—C9A—C8A	107.0 (8)	C11—Hf1—C14	82.4 (2)
C10A—C9A—Hf1	73.3 (5)	C11—Hf1—C2	136.1 (3)
C8A—C9A—Hf1	72.2 (5)	C14—Hf1—C2	111.5 (2)
C10A—C9A—H9A	126.5	C11—Hf1—C8A	133.6 (3)
C8A—C9A—H9A	126.5	C14—Hf1—C8A	99.6 (3)
Hf1—C9A—H9A	119.9	C2—Hf1—C8A	86.7 (3)
C9A—C10A—C6A	109.6 (8)	C11—Hf1—C3	109.7 (2)
C9A—C10A—Hf1	74.3 (5)	C14—Hf1—C3	135.8 (2)
C6A—C10A—Hf1	73.7 (5)	C2—Hf1—C3	32.41 (9)
C9A—C10A—H10A	125.2	C8A—Hf1—C3	100.8 (3)
C6A—C10A—H10A	125.2	C11—Hf1—C7A	119.2 (3)
Hf1—C10A—H10A	118.6	C14—Hf1—C7A	131.0 (3)
C7B—C6B—C10B	104.8 (16)	C2—Hf1—C7A	83.8 (3)
C7B—C6B—Hf1	73.8 (11)	C8A—Hf1—C7A	32.78 (13)
C10B—C6B—Hf1	75.8 (15)	C3—Hf1—C7A	81.2 (3)
C7B—C6B—H6B	127.6	C11—Hf1—C6B	105.3 (5)
C10B—C6B—H6B	127.6	C14—Hf1—C6B	138.4 (5)
Hf1—C6B—H6B	115.4	C2—Hf1—C6B	91.3 (6)
C6B—C7B—C8B	110.6 (15)	C3—Hf1—C6B	81.0 (5)
C6B—C7B—Hf1	73.7 (12)	C11—Hf1—C7B	135.4 (4)
C8B—C7B—Hf1	75.1 (13)	C14—Hf1—C7B	118.7 (5)
C6B—C7B—H7B	124.7	C2—Hf1—C7B	75.7 (4)
C8B—C7B—H7B	124.7	C3—Hf1—C7B	83.0 (4)
Hf1—C7B—H7B	118.2	C6B—Hf1—C7B	32.4 (4)
C7B—C8B—C9B	106.8 (15)	C11—Hf1—C4	82.7 (2)
C7B—C8B—Hf1	72.5 (12)	C14—Hf1—C4	116.4 (2)
C9B—C8B—Hf1	75.3 (14)	C2—Hf1—C4	53.5 (2)
C7B—C8B—H8B	126.6	C8A—Hf1—C4	133.1 (3)
C9B—C8B—H8B	126.6	C3—Hf1—C4	32.30 (9)
Hf1—C8B—H8B	117.7	C7A—Hf1—C4	110.0 (3)
C10B—C9B—C8B	107.0 (15)	C6B—Hf1—C4	105.2 (5)
C10B—C9B—Hf1	73.5 (15)	C7B—Hf1—C4	114.9 (4)
C8B—C9B—Hf1	72.5 (14)	C11—Hf1—C6A	87.0 (3)
C10B—C9B—H9B	126.5	C14—Hf1—C6A	126.7 (3)
C8B—C9B—H9B	126.5	C2—Hf1—C6A	112.1 (3)
Hf1—C9B—H9B	119.4	C8A—Hf1—C6A	54.5 (3)
C9B—C10B—C6B	110.7 (16)	C3—Hf1—C6A	96.9 (3)
C9B—C10B—Hf1	74.9 (15)	C7A—Hf1—C6A	32.62 (13)
C6B—C10B—Hf1	71.7 (14)	C4—Hf1—C6A	113.8 (3)
C9B—C10B—H10B	124.7	C11—Hf1—C10A	79.8 (3)
C6B—C10B—H10B	124.7	C14—Hf1—C10A	94.2 (3)
Hf1—C10B—H10B	120.3	C2—Hf1—C10A	136.5 (3)
C12—C11—Hf1	108.6 (4)	C8A—Hf1—C10A	53.8 (3)
C12—C11—H11A	110.0	C3—Hf1—C10A	129.3 (3)
Hf1—C11—H11A	110.0	C7A—Hf1—C10A	53.5 (3)
C12—C11—H11B	110.0	C4—Hf1—C10A	142.2 (3)
Hf1—C11—H11B	110.0	C6A—Hf1—C10A	32.51 (13)
H11A—C11—H11B	108.3	C11—Hf1—C1	119.2 (2)
C15—C12—C11	113.9 (6)	C14—Hf1—C1	83.3 (2)
C15—C12—C13	115.7 (6)	C2—Hf1—C1	32.22 (9)
C11—C12—C13	109.4 (5)	C8A—Hf1—C1	107.1 (3)
C15—C12—H12	105.6	C3—Hf1—C1	53.3 (2)
C11—C12—H12	105.6	C7A—Hf1—C1	114.5 (3)
C13—C12—H12	105.6	C6B—Hf1—C1	123.4 (6)
C17A—C13—C14	113.7 (5)	C7B—Hf1—C1	102.9 (4)
C17B—C13—C14	111.0 (9)	C4—Hf1—C1	53.0 (2)
C17A—C13—C12	112.5 (5)	C6A—Hf1—C1	144.3 (3)
C17B—C13—C12	129.3 (10)	C10A—Hf1—C1	160.1 (3)
C14—C13—C12	113.1 (5)		
			
C5—C1—C2—C3	1.2 (8)	C10B—C6B—C7B—Hf1	−70.1 (18)
Hf1—C1—C2—C3	67.1 (5)	C6B—C7B—C8B—C9B	2 (3)
C5—C1—C2—Hf1	−66.0 (5)	Hf1—C7B—C8B—C9B	68.1 (18)
C1—C2—C3—C4	−0.8 (8)	C6B—C7B—C8B—Hf1	−65.7 (15)
Hf1—C2—C3—C4	67.2 (5)	C7B—C8B—C9B—C10B	0 (3)
C1—C2—C3—Hf1	−67.9 (5)	Hf1—C8B—C9B—C10B	66 (2)
C2—C3—C4—C5	0.0 (8)	C7B—C8B—C9B—Hf1	−66.1 (16)
Hf1—C3—C4—C5	66.7 (5)	C8B—C9B—C10B—C6B	−2 (3)
C2—C3—C4—Hf1	−66.7 (5)	Hf1—C9B—C10B—C6B	63 (2)
C3—C4—C5—C1	0.7 (7)	C8B—C9B—C10B—Hf1	−65.3 (19)
Hf1—C4—C5—C1	66.6 (4)	C7B—C6B—C10B—C9B	3 (3)
C3—C4—C5—Hf1	−66.0 (5)	Hf1—C6B—C10B—C9B	−65 (2)
C2—C1—C5—C4	−1.2 (7)	C7B—C6B—C10B—Hf1	68.7 (15)
Hf1—C1—C5—C4	−66.3 (4)	Hf1—C11—C12—C15	−168.5 (5)
C2—C1—C5—Hf1	65.2 (5)	Hf1—C11—C12—C13	−37.3 (6)
C10A—C6A—C7A—C8A	−1.3 (11)	C15—C12—C13—C17A	−46.1 (8)
Hf1—C6A—C7A—C8A	65.5 (7)	C11—C12—C13—C17A	−176.3 (6)
C10A—C6A—C7A—Hf1	−66.8 (6)	C15—C12—C13—C17B	−28.1 (13)
C6A—C7A—C8A—C9A	2.0 (11)	C11—C12—C13—C17B	−158.3 (10)
Hf1—C7A—C8A—C9A	68.2 (7)	C15—C12—C13—C14	−176.6 (5)
C6A—C7A—C8A—Hf1	−66.2 (7)	C11—C12—C13—C14	53.2 (7)
C7A—C8A—C9A—C10A	−1.8 (11)	C17A—C13—C14—Hf1	−169.1 (5)
Hf1—C8A—C9A—C10A	65.5 (7)	C17B—C13—C14—Hf1	166.4 (10)
C7A—C8A—C9A—Hf1	−67.3 (7)	C12—C13—C14—Hf1	−39.2 (6)
C8A—C9A—C10A—C6A	1.0 (11)	C11—C12—C15—C16	67.2 (9)
Hf1—C9A—C10A—C6A	65.8 (6)	C13—C12—C15—C16	−60.9 (9)
C8A—C9A—C10A—Hf1	−64.8 (7)	C17B—C13—C17A—C18A	37.4 (19)
C7A—C6A—C10A—C9A	0.2 (10)	C14—C13—C17A—C18A	−50.7 (10)
Hf1—C6A—C10A—C9A	−66.2 (7)	C12—C13—C17A—C18A	179.1 (7)
C7A—C6A—C10A—Hf1	66.4 (7)	C17A—C13—C17B—C18B	−61 (2)
C10B—C6B—C7B—C8B	−4 (3)	C14—C13—C17B—C18B	40 (3)
Hf1—C6B—C7B—C8B	66.6 (16)	C12—C13—C17B—C18B	−109 (2)
