# Supplementary figures and images for: Crystal structure of bis­(η5-cyclo­penta­dien­yl)(2,3-di­ethyl­butane-1,4-di­yl)hafnium(IV)
Source: Acta Crystallogr E Crystallogr Commun. 2015 Jan 1;71(Pt 1):m7. doi: 10.1107/S2056989014026929 (PMC4331853; doi:10.1107/S2056989014026929)

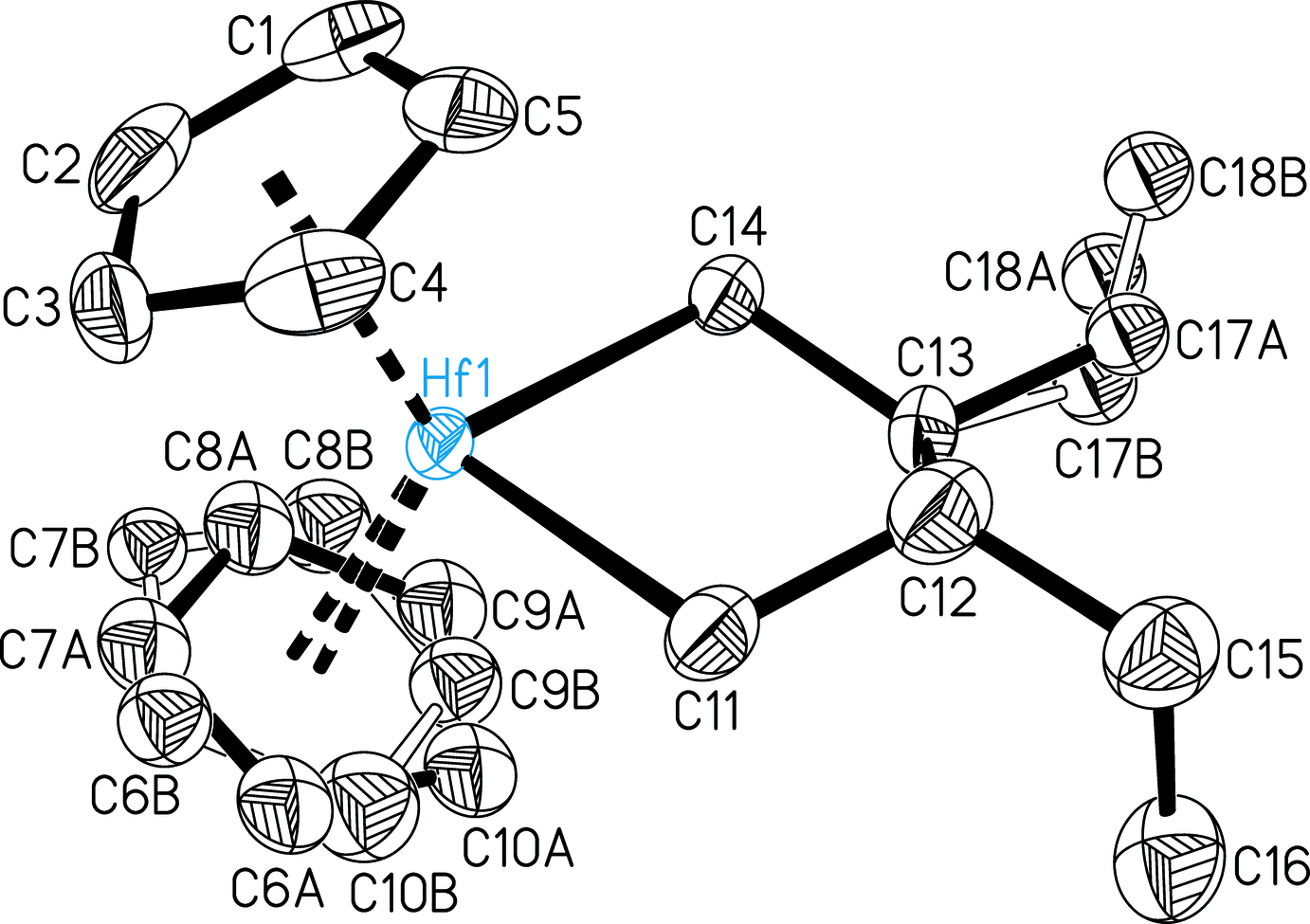

Supplement: Supplementary file 3 [file e-71-000m7-fig1.tif]
